# BRD4 Inhibition by AZD5153 Promotes Antitumor Immunity via Depolarizing M2 Macrophages

**DOI:** 10.3389/fimmu.2020.00089

**Published:** 2020-02-28

**Authors:** Xi Li, Yu Fu, Bin Yang, Ensong Guo, Yifan Wu, Jia Huang, Xiaoxiao Zhang, Rourou Xiao, Kezhen Li, Beibei Wang, Junbo Hu, Chaoyang Sun, Gang Chen

**Affiliations:** ^1^Department of Gynecology and Obstetrics, Tongji Medical College, Tongji Hospital, Huazhong University of Science and Technology, Wuhan, China; ^2^Department of Gynecology and Obstetrics, The Central Hospital of Wuhan, Wuhan, China; ^3^Department of Gastrointestinal Surgery, Tongji Medical College, Tongji Hospital, Huazhong University of Science and Technology, Wuhan, China

**Keywords:** high-grade serous ovarian cancer (HGSOC), tumor microenvironment, tumor-associated macrophages, M2 macrophage, macrophage repolarization, BRD4 inhibitor

## Abstract

High-grade serous ovarian cancer (HGSOC), with its high recurrence rates, urges for reasonable therapeutic strategies that can prolong overall survival. A tumor microenvironment (TME) discloses prognostic and prospective information on cancer, such as the expression level of PD-1 or PD-L1. However, in HGSOC, the impact of the therapies aiming at these targets remains unsatisfying. Tumor-associated macrophages (TAMs) in HGSOC make up a large part of the TMEs and transform between diverse phenotypes under different treatments. AZD5153 inhibiting BRD4, as a potential therapeutic strategy for HGSOC, was demonstrated to confer controversial plasticity on TAMs, which shows the need to uncover its impact on TAMs in HGSOC. Therefore, we established models for TAMs and TAMs co-culturing with T lymphocytes *in vitro*. Via RT-PCR and flow cytometry, we find that AZD5153 resets TAMs from M2-type macrophages to M1-like macrophages, consequently promoting pro-inflammatory cytokine secretion and thus activating CD8^+^ cytotoxic T lymphocytes (CTLs) *in vitro*. This modification occurs on MAF transcripts in TAMs and modified by BRD4, which is the target of AZD5153. Importantly, the 3-D microfluid model demonstrates that AZD5153 sensitizes ovarian cancer to anti-PD-L1 therapy. Our results clarify that besides eliminating tumor cells directly, AZD5153 works as a regulator of the TAM phenotype, providing a novel strategy combining AZD5153 and PD-1/PD-L1 antibody that could benefit HGSOC patients.

## Introduction

High-grade serous ovarian cancer (HGSOC) is an insidious and aggressive disease in aging women, which is the fifth leading cause of mortality in US women. Only 14.9% of HGSOCs are diagnosed at an early stage, whereas nearly 60% are diagnosed at an advanced stage with apparent symptoms caused by ascites and peritoneal metastases. Even worse, the 5-year survival rate for the advanced stage is <50% (1). A comprehensive view of peritoneal metastasis is essential for developing a therapeutic strategy and ultimately improving the prognosis.

Since the development of sequencing technology, tumor microenvironments (TME) of solid tumors were gradually elucidated by the deconvolution of sequencing data, including the tumor-associated macrophages (TAMs). As for ovarian cancer, it has emerged that macrophages are indispensable in metastasis due to the formation of spheroids with themselves as the core and free cancer cells detached from the primary tumor (2). Macrophages derive along a spectrum between anti-tumorigenic (M1-like) macrophages and pro-tumorigenic (M2-like) macrophages polarized by the TME and extracellular signals. M1-like macrophages promote local immunity by secreting a variety of cytokines, such as IL-12, IFN-γ, and TNF-α (3). These acute inflammatory mediators, particularly, IL-12 as well as its family member IL-23, upregulate Th1-like immune response and consequently activate the destructive effect of cytotoxic T lymphocytes (4). On the contrary, M2-like macrophages contribute to tumor progression by enriching anti-inflammatory cytokines in tumor TME, such as IL-10 and TGF-β, which typically promote immunosuppression, cancer cell proliferation, invasion, and metastasis (5). In general, increasing M2-like macrophages are correlated with advanced stage and poor outcome in epithelial ovarian cancer (6). Conversely, high M1/M2 macrophage ratios in tumor tissues associated with long-term prognosis (7, 8). The bipolarization and flexibility of TAMs denote that converting M2-like macrophages to M1-like tumoricidal ones facilitates promising therapeutic benefits.

Macrophage alternative polarization was predominantly controlled by a repertoire of lineage-determining transcription factors (TFs) (9). Recent studies reported that M2-like and M1-like macrophage polarization was regulated by two exclusive sets of TFs, respectively. Besides inhibiting the target, the inhibitors of one of the lineage-determining TFs also modulate its M2-like gene sets (10). In other words, the phenotype-specific gene expression patterns can be epigenomically rearranged under different conditions even if these macrophages are in terminal forms (11). In contrast to the abundance of studies about M2-like enhancer induction, little is known about the phenotype changes when deactivating enhancers by a specific epigenomic inhibitor.

Bromodomain containing 4 (BRD4), a member of the bromodomain and extraterminal (BET) protein family, interacts with the acetylated lysine residues of histone tails on chromatins and consequently promotes several oncogenic transcriptions such as c-MYC, which contributes to cancer cell proliferation (12). The inhibitor of BET shows beneficial effects in preclinical cancer models with hematological and solid tumors, which is attributed to its selective downregulation of targeted and context-dependent gene expression (13, 14). The Cancer Genome Atlas (TCGA) data illustrate that BRD4 amplification occurs most frequently in HGSOC patients across all represented cancer subtypes (15), which implies that HGSOC patients is potential demographic that will benefit from BRD4 inhibitors. Given the fact that inflammatory gene expression is tightly controlled through a chromatin “reader,” past studies have been devoted to revealing the function of BET proteins involved in the inflammatory response. Knocking down BRD2, another member of the BET protein family, has been proven to prevent pro-inflammatory cytokine production including TNF-a, IL-6, and MCP-1 in macrophages (16). One controversial finding is that BET inhibition suppresses PD-L1 expression of both immune and tumor cells in mice, which is correlated with a warmer antitumor immune response and more restricted tumor growth (17). The conflict phenomenon of BET inhibitor effects in TME remains to be further discussed.

Accordingly, to evaluate the global effect of AZD5153, a novel, potent, and specific BRD4 inhibitor (18) in the TME of ovarian cancer, we profiled BET inhibitory effects on TAMs *in vitro* and *in vivo*. Our findings suggest that BET inhibitor contributes to tumoricidal effects through both direct killing effects and tumor immune response activation. This antitumor immunity is attributed to the suppression of BRD4 at the MAF binding site in macrophages. Thus, AZD5153 disassembled BRD4 to suppress MAF and its M2-like gene expression, which indicates the importance of MAF in the M2 gene landscape since it can be targeted by clinical therapies. Also, we further confirmed that BET inhibition sensitizes ovarian cancer to immune checkpoint inhibitory therapy in the 3-D microfluid model.

## Results

### AZD5153 Scaled Down M2-Like Macrophages in Solid Tumors

**Figure 1:**
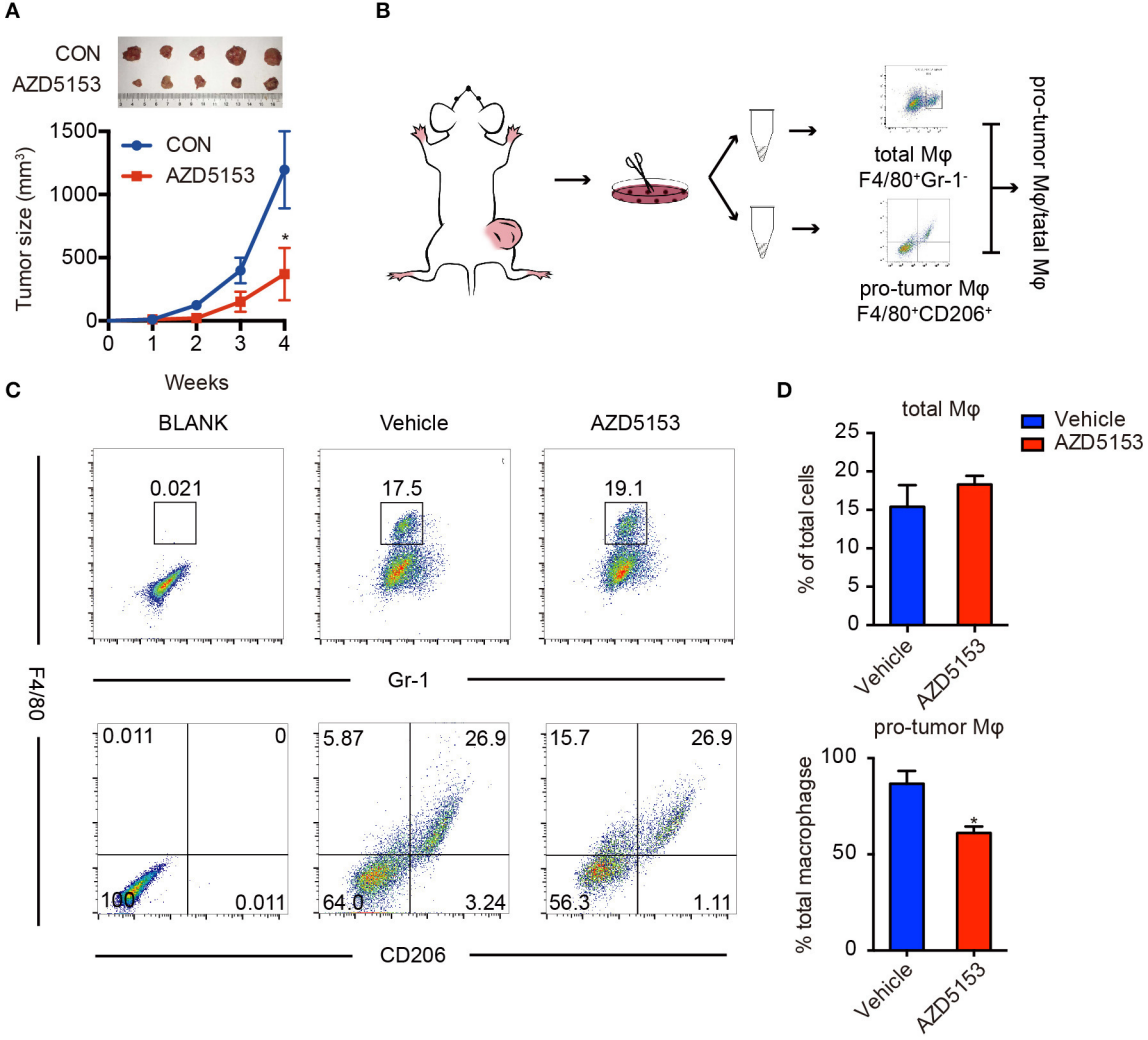
AZD5153 scaled down M2-like macrophages in solid tumor. **(A)** Representative images and tumor volume measurements of CT26 tumors in BALB/c mice with continuous treatment (*n* = 5); volume of tumor was measured every week: *V* = (*L* × *W*^2^)/2. The mice were treated with AZD5153 (0.5 mg/ml) or vehicle 100 μl/day by gavage for 4 weeks. **(B)** Diagram for flow cytometry experiments. Tumors from the same group were dissociated and equally divided into two Eppendorf tubes for flow cytometry analysis. **(C)** Flow cytometry analysis of total macrophages (top) and M2-type macrophages (bottom) in CT26 tumors after treatments. **(D)** Bar chart for **(C)**. Data represent mean ± SEM. *P < 0.05.

To determine whether AZD5153 disturbs the dynamical equilibrium of TME, we established a solid tumor mouse model by subcutaneously injecting CT26 cells into the right lower limbs of BALB/c female recipient mice. Solid tumors were visible on the right lower limbs of all the mice in 7 days. The mice were randomly divided into two groups and orally treated with AZD5153 or vehicle, respectively, for 4 weeks. After the 4-week treatment, AZD5153 significantly inhibited tumor growth (Figure 1A). The proportions of total TAMs and M2-like macrophages were analyzed by flow cytometry separately (Figure 1B). We found that M2-like macrophage proportion in total macrophages was significantly shrunk by AZD5153, while the total macrophage proportion in whole solid tumor cells was unchanged (Figures 1C,D). These results indicated that AZD5153 did not affect the total number of macrophages among the TME but modified the proportion of each macrophage phenotype *in vivo*.

### AZD5153 Depolarized the Pro-tumor Phenotype of Macrophage *in vitro*

To investigate the exact effects of AZD5153 on macrophages, we established an *in vitro* macrophage culture model simulated with TME *in vivo*. It has been shown that IL-4 and IL-13, in tandem with PMA, induce THP-1 cells polarizing to M2-like macrophages *in vitro*, and IL-4 alone is adequate to achieve the induction for RAW264.7 cells (19). We cultured inactivated THP-1 and RAW264.7 with a conditional medium (CM) which was harvested from the supernatant of human and mouse ovarian cancer cell lines, respectively, or the cytokines mentioned above for 48 h. Macrophage cell lines were efficiently induced to an anti-inflammatory M2-like phenotype by the CM with a significantly reversed IL-10/IL-12 expression ratio compared with the inactivated ones (Supplementary Figures 1A,B). In conclusion, the ovarian cancer cell CM was adequate to transform inactivated macrophages to an M2-like phenotype *in vitro*.

**Figure 2:**
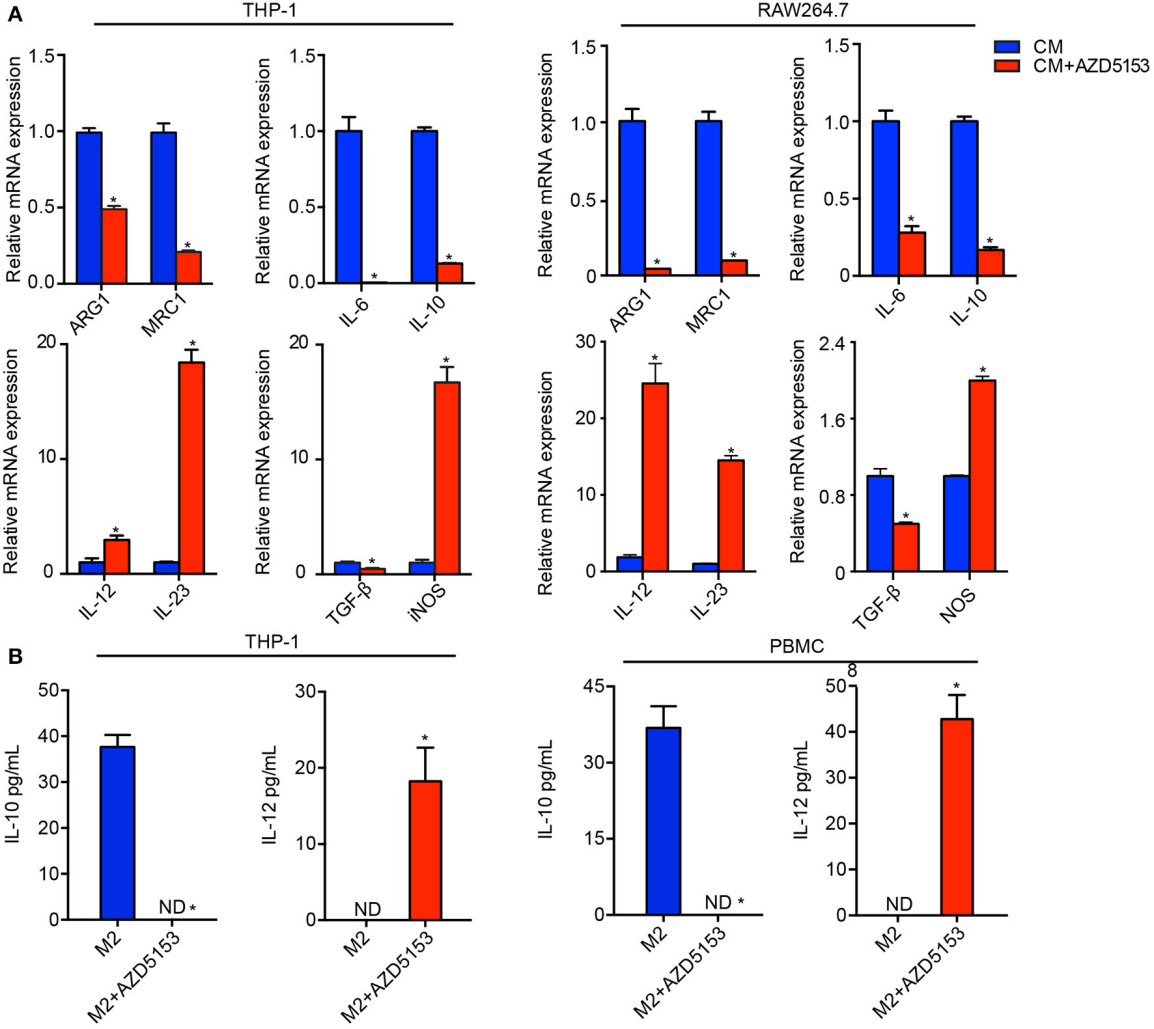
AZD5153 reversed the pro-tumor phenotype of macrophages *in vitro*. **(A)** qPCR evaluation of ARG1, MRC1, and relative cytokines in THP-1 and RAW264.7 macrophages that were separately induced by conditional medium (CM) originating from human and mouse ovarian cancer cells (*n* = 3). Data represent mean ± SEM. *P < 0.05. **(B)** ELISA evaluation of IL-10 and IL-12 for M2 macrophages treated with and without AZD5153. Left: experiments in THP-1 (*n* = 3). Right: experiments for healthy female peripheral blood macrophages (*n* = 5). Data represent mean ± SEM. *P < 0.05.

Taking advantage of the cell model above, we directly treated the M2-like macrophages with AZD5153 *in vitro*. M2-like macrophage markers were significantly abrogated by AZD5153, along with those cytokines involved in immune tolerance and immune evasion, such as IL-6, IL-10, and TGF-β (20). The similar results occurred when it came to murine bone marrow-derived macrophages (Supplementary Figure 3C). On the other hand, iNOS, an M1 marker associated with the activated immune response of macrophages (21), was clearly upregulated by AZD5153. IL-12 and its family member IL-23 (22, 23) were also consistently and significantly increased by AZD5153 (Figure 2A). In order to validate the stability of gene expression under the experimental conditions in this study (24), we used three different normalizers and finally ascertained the results definitely (Supplementary Figure 3). We further verified the concentration of IL-10 and IL-12 in the supernatant of macrophages by ELISA and found that the ratio of IL-10/IL-12 was significantly reversed in AZD5153-treated supernatant compared with one of M2-like macrophages (Figure 2B). However, M2-like macrophages treated by AZD5153 only partially transform to an M1-like phenotype due to the unchanging expression levels of IFN-γ and TNF-α (Supplementary Figure 2) (25, 26). Accordingly, AZD5153 reversed the M2 phenotype induced by CM and promoted M1-like transformation, which indicated that AZD5153 potentially activated antitumor immune response by depolarizing M2-like macrophages.

### AZD5153 Facilitated Macrophages' Capacity to Activate CD8^+^ T Cell *in vitro*

**Figure 3:**
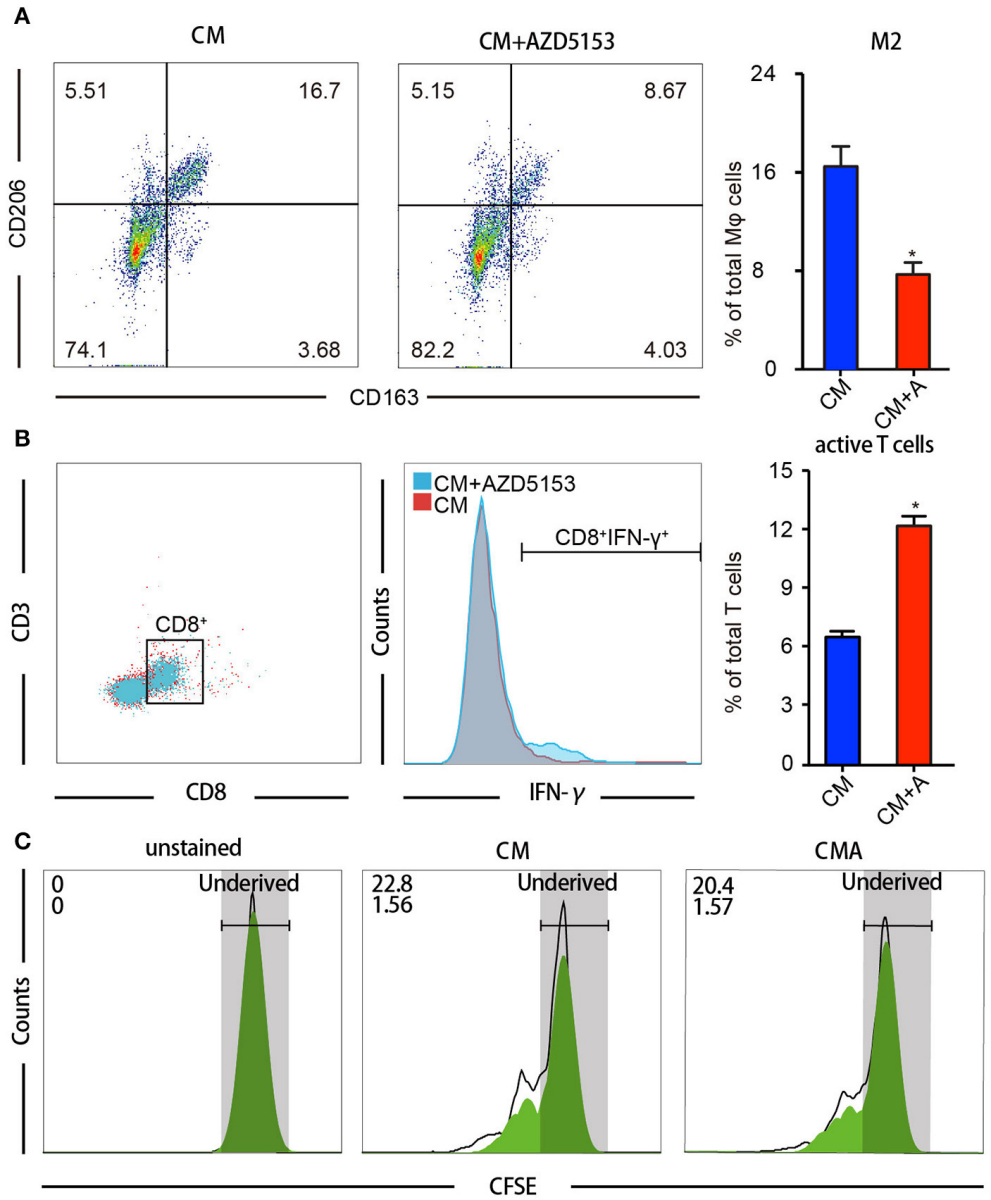
AZD5153 facilitates macrophages' capacity to activate CD8^+^ T cells *in vitro*. T cells isolated from female volunteers' peripheral blood were co-cultured with drug-treated macrophages separated from the same peripheral blood. **(A)** Flow cytometry analysis for the percentage of CD206^+^CD163^+^ macrophages among CM-induced macrophages with and without treatment of AZD5153 (*n* = 3). **(B)** Flow cytometry analysis for the percentage CD8^+^IFNγ^+^ T cells co-cultured with macrophages in **(A)**. **(C)** Cell division index of CD8^+^ T cells by CFSE assays (*n* = 3). Data represent mean ± SEM. *P < 0.05.

We further investigated whether AZD5153-treated TAMs could strengthen the antitumor effects of CD8^+^ cytotoxic T cells. Within TME, antitumor immunity depends chiefly on CD8^+^ cytotoxic T lymphocytes (27). Primary M2-like macrophages treated with or without AZD5153 (Figure 2A) were co-cultured with T cells separated from the same donor for another 48 h. Flow cytometry analysis revealed that M2-like macrophages treated with AZD5153 recover the capacity to upregulate the proportion of IFN-γ^+^T in CD8^+^T cells *in vitro* (Figures 3A,B).

Besides the activation of T cell, the proliferation of the T cell also defines the strength of antitumor immunity (28). Accordingly, we detected the cell division index of CD8^+^ T cells by carboxyfluorescein succinimidyl ester (CFSE) assays and observed that AZD5153-treated M2-like macrophages neither promoted nor inhibited CD8^+^ T cell proliferation (Figure 3C). Here, we concluded that AZD5153-treated M2-like macrophages restored the capacity to activate CD8^+^ T cells *in vitro*, but not promote the proliferation.

### AZD5153 Depolarized M2-Like Macrophages by Inhibiting MAF

**Figure 4:**
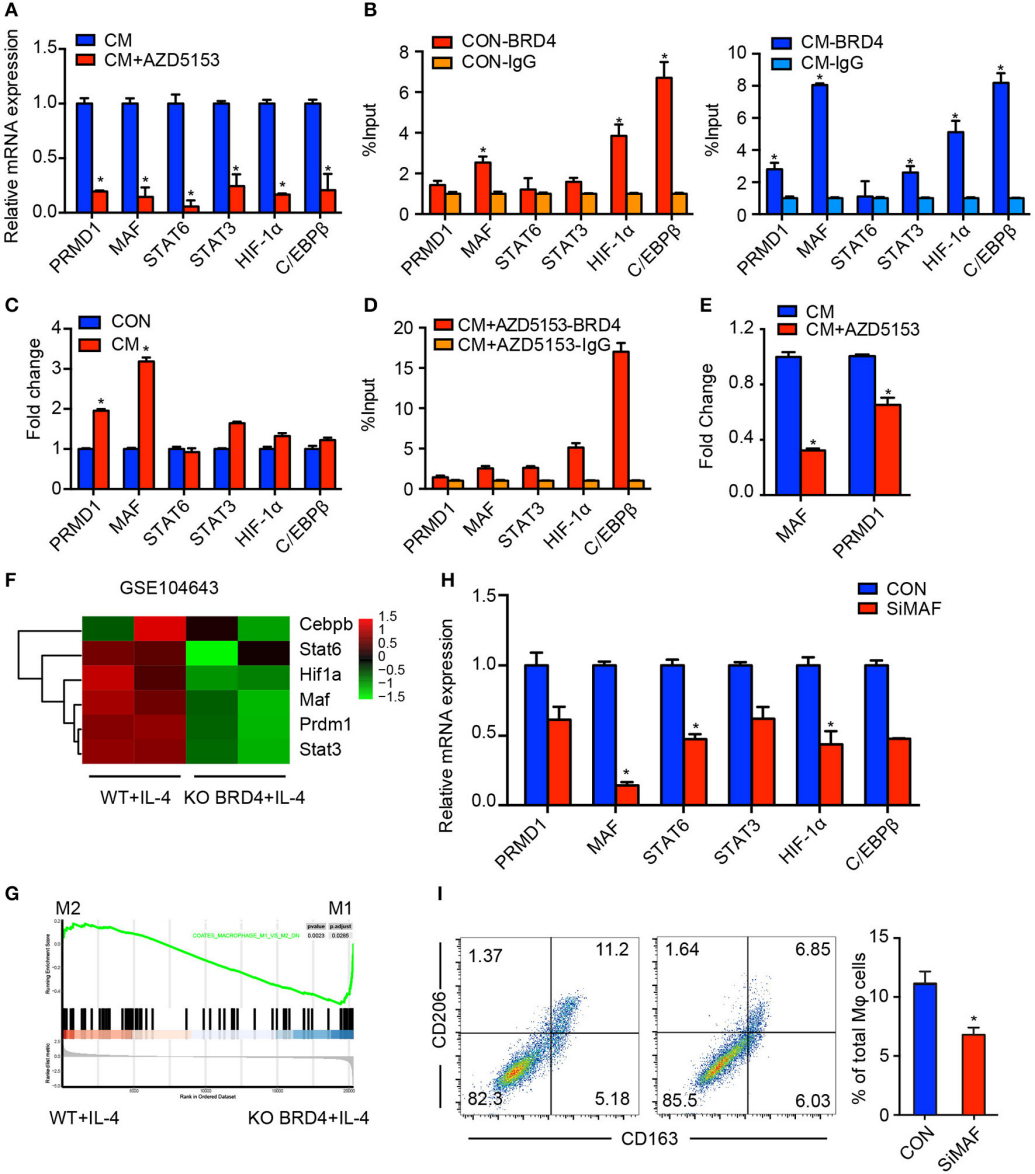
AZD5153 depolarizes M2-like macrophages by inhibiting MAF. **(A)** qPCR evaluation of transcription factors including PRMD1, MAF, STAT6, STAT3, HIF-1α, and C/EBPβ in CM-induced macrophages treated with and without AZD5153. **(B,C)** ChIP-PCR evaluation of binding events between each transcription factor and BRD4 in macrophages induced with and without CM. **(D)** ChIP-PCR evaluation of binding events between each transcription factor and BRD4 in macrophages induced with CM and treated with AZD5153. **(E)** ChIP-PCR evaluation of binding events between BRD4 and two transcription factors MAF and PRMD1 in CM-induced macrophages treated with and without AZD5153. **(D)** The mRNA expressions of six transcription factors in **(A)** were analyzed by qPCR in MAF and control (NC) siRNA in CM-induced macrophages. **(F)** The differential analysis of six transcription factors between the wild-type mouse model and BRD4-knockout mouse model under the stimulation of IL-4 (GSE104643). **(G)** The GSEA analysis of the same dataset in **(E)**. **(H)** The mRNA expression of six transcription factors in **(A)** were analyzed by qPCR in MAF and control (NC) siRNA in CM-induced macrophages. **(I)** Flow cytometry analysis for the percentage of CD206^+^CD163^+^ macrophages among total macrophages. Left: diagram for flow cytometry analysis. Right: bar chart for flow cytometry analysis (*n* = 3). Data represent mean ± SEM. *P < 0.05.

Several TFs have been demonstrated to be involved in modulating the M2 phenotype of macrophages, such as PRDM1, MAF, STAT6, STAT3, HIF-1α, and C/EBPβ (10, 29–33). We assumed that AZD5153 epigenetically regulated the macrophage phenotype via alternating the expression levels of pivotal TFs. Therefore, we detected the expression levels of six crucial TFs involved in the M2-like phenotype with or without AZD5153 treatment. Intriguingly, all of them were downregulated by AZD5153 (Figure 4A). Since AZD5153 specifically targeted BRD4 bromodomains in pharmacology, we consequently screened the target TFs by focusing on the ones regulated by BRD4 (34). As evidenced by CHIP-PCR, we found that the TF binding site (TFBS) of five TFs had binding events to BRD4 after it was cultured with or without CM of ovarian cancer cells (Figure 4B), among which PRDM1 and MAF had increasing binding events after CM treatment (Figure 4C). Accordingly, these two TFs could be key TFs that regulate macrophage phenotype related to ovarian cancer. Moreover, the GEO dataset (GSE1046431) revealed that genetic deletion of BRD4 led to the reduction of MAF and PRDM1 and impeded the polarization toward M2-type macrophages (Figures 4F,G). In this study, AZD5153 only significantly reduced the binding of BRD4 to MAF TFBS along with a slight reduction to PRDM1 TFBS, but with no statistical significance (Figures 4D,E). MAF markedly downregulated those TFs mentioned above, such as STAT6 and HIF-1α, partially reduced others, and successfully depolarized M2-like macrophages (Figures 4H,I). Consequently, AZD5153 reversed the phenotype of M2-like macrophages by inhibiting the expression of MAF.

### AZD5153 Sensitizes Ovarian Cancer to αPD-L1

**Figure 5:**
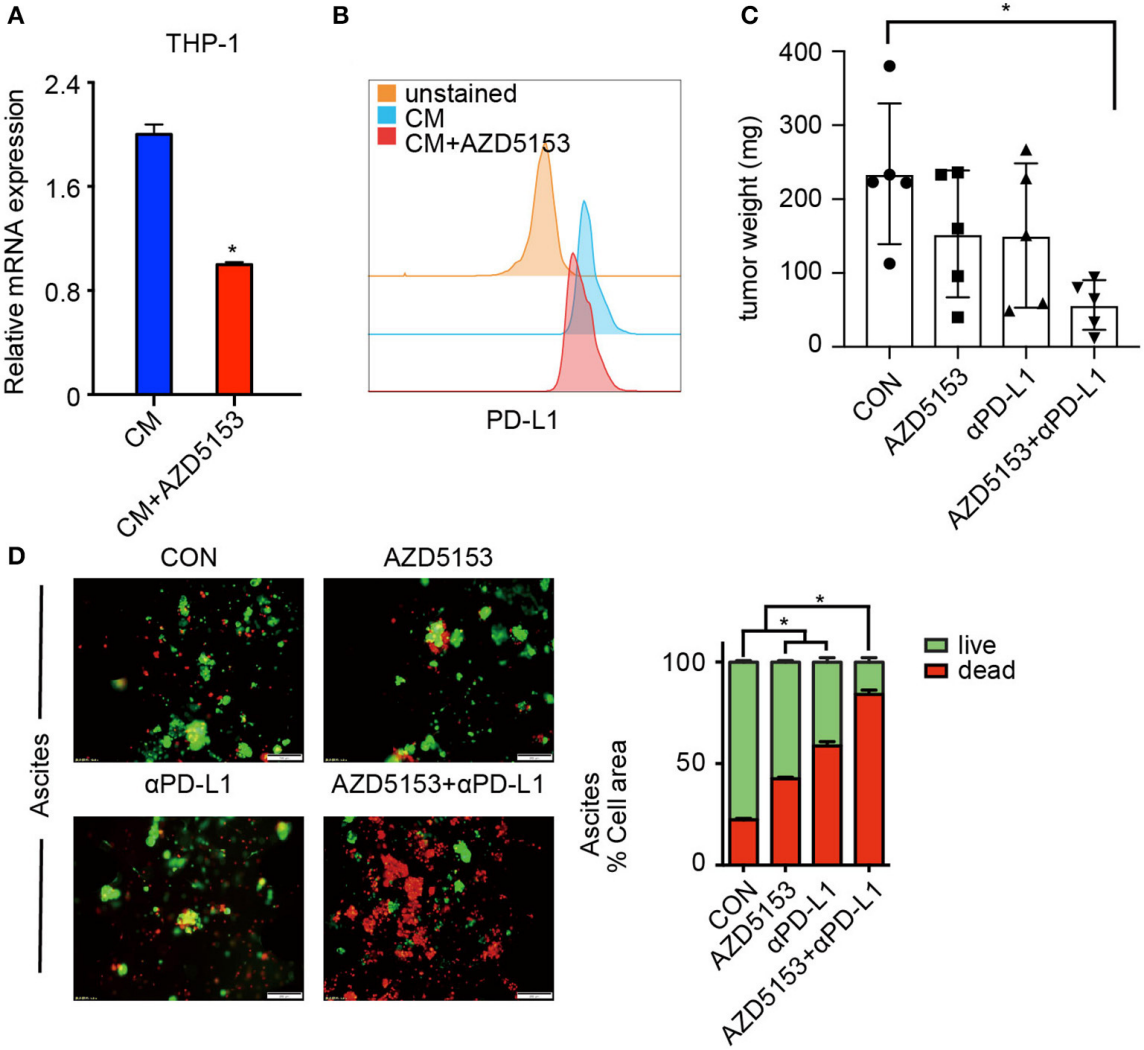
AZD5153 sensitizes ovarian cancer to αPD-L1. **(A,B)** Expression of PD-L1 in CM-induced THP-1 macrophages treated with and without AZD5153 analyzed with qPCR and flow cytometry (*n* = 3). **(C)** The total weights of separate tumors in each mouse were calculated and displayed according to the group (*n* = 5). **(D)** Tumor microspheres isolated from ascites of an untreated ovarian cancer patient in 3-dimensional microfluidic chips. Left: diagram of microspheres under treatment with no drug, single drug (AZD5153 or αPD-L1) and combinational strategy. Right: bar chart demonstrating the percentage of live and dead microspheres (*n* = 3). Data represent mean ± SEM. *P < 0.05.

TAMs demonstrated a primary expression of PD-L1 in ovarian carcinoma, and patients with high PD-L1 expression levels have significantly worse survival than cases of low PD-L1 expression (35). We further accessed the expression of PD-L1 on AZD5153-treated M2-like macrophages. As expected, AZD5153 downregulated PD-L1 expression increased by CM (Figures 5A,B). Thus, we hypothesized that AZD5153 probably sensitized ovarian cancer to anti-PD-L1 therapy. To evaluate the therapeutic effects *in vivo*, we established an ovarian cancer mouse model by intraperitoneally injecting ID8 cells into C57BL/6 female recipient mice. The tumor weights and the quantity of tumor cells in mice treated with combinational therapy were significantly less than those in the control group (Figure 5C and Supplementary Figure 4A). There was no significant difference in the mouse weight among four groups, which indicated the safety of the therapy (Supplementary Figure 4B).

A study on 3-D microfluidic culture shows that patient-derived organotypic tumor spheroids that are isolated from human tumors retain an autologous immune environment in 3-D culture chips (36). We cultured tumor microspheres from ascites of an untreated ovarian cancer patient in 3-D microfluidic chips. Both AZD5153 and αPD-L1 kill tumor microspheres effectively. Additionally, the microspheres treated with the combination of AZD5153 and αPD-L1 displayed a striking death and destruction compared to groups treated with either drug alone (Figure 5D). Furthermore, we collected carcinoma blocks, digested them into tumor microspheres, and dealt them with different therapeutic strategies in 3-D chips. The synergistic activity of combination drug therapy was analogously observed in primary ovarian carcinoma samples, even though it was not observed as strikingly as in ascites (Supplementary Figure 4C). In summary, AZD5153 sensitized ovarian cancer to αPD-L1 for primary sites as well as in ascites.

## Discussion

Communication between macrophages and tumor cells leads to egress and invasion of tumor cells in ovarian cancer, which makes TAMs a crucial target for cancer therapy (37). BRD4, a member of the BET protein family, amplifies oncogenic transcription by recruiting transcriptional machinery or indirectly by binding to enhancers, contributing to cancer cell proliferation and survival (38). We demonstrated that decreased MAF transcription appears to be a significant contributor to the effects of AZD5153 on M2-like macrophage depolarization and to be sufficient for much antitumor immune response activation.

In the microenvironment of solid tumors, TAMs are in tumor-educated forms associated with tumor progression and malignancy (39). However, BET inhibition has previously been associated with resisting LPS-induced inflammatory response in atherogenesis, osteoporosis, and COPD (40–42), pointing to an immune modulatory effect on the inflammatory response. Intriguingly, research for pancreatic cancer observed that BET inhibition impeded pro-inflammatory pathways, while it also had the capacity to skew human macrophage toward M1 macrophages (16). Given these disputed findings, we provided the first evidence that AZD5153 skews M2-type macrophages toward M1-like macrophages *in vivo* and *in vitro*, which indicates the pro-inflammatory capability of AZD5153.

The underpinning mechanism of BET inhibitors regulating macrophage phenotypes has been explored in the past 5 years. It has been demonstrated that the regulatory function of BET inhibition in the inflammatory response was attributed to decreases in NF-κB that were assessed at the RNA level. However, no significant difference in the expression of NF-κB was observed in our hands. The pronounced expression of NF-κB in M1 macrophages, rather than M2 macrophages, may explain the differential target of BET inhibition between the inflammatory response and anti-inflammatory response. Here, in M2 macrophages, we showed that AZD5153 significantly reduced the binding of BRD4 to MAF TFBS among several TFs. The depletion of MAF exclusively in M2 macrophages is sufficient to induce the repolarization of M2, mimicking AZD5153. Taken together, in M2 macrophages induced by ovarian cancer, MAF rather than NF-κB appears to be a vital component of the process by which BRD4i causes repolarization of M2 macrophages.

Several publications reported that sufficient M2-type macrophages that make depletion occur by blocking CSF-1R antibodies are adequate to suppress tumor growth in tumor models (43, 44). Instead of M2 depletion, AZD5153 skews M2 macrophages to M1 macrophages and turns over secretion of cytokines with increasing IL-12 and decreasing IL-10 in the TAM module. Also, a therapy that combines M2 depletion with adoptive T-cell transfer showed significant tumor growth delay (43). We demonstrated that AZD5153-repolarized macrophages augmented activation of CD8^+^ CTLs *in vitro*. Taken together, AZD5153 seems to be a better choice for macrophage-targeted therapy in ovarian cancer. However, apart from a substantial increase in activated CD8^+^ T cells, we did not observe a significant change in proliferation of CD8^+^ CTLs in response to AZD5153-treated M2 macrophages in our model, which indicates that the increasing scale of CD8^+^ CTLs are from direct T-cell activation by increasing pro-inflammatory M1 macrophages. A mouse model of ovarian cancer shows that BET inhibition limits tumor progression in a CD8^+^ cytotoxic T-cell-dependent manner (17). Further studies will be required to determine the effects of AZD5153-treated M2 macrophages on CD8^+^ T lymphocytes *in vivo* and the precise role that they play in the antitumor efficacy of AZD5153.

Although αPD-L1 has recently achieved impressive success in the treatment of different cancer types, the majority of ovarian cancer patients do not derive benefit from this treatment (45). Expression of PD-L1 in tumor-infiltrating macrophages is correlated with clinical responses to αPD-L1 treatment in several types of cancer (46). Notably, in BET inhibitor-treated mice, a significant decrease in PD-L1 expression was observed in macrophages isolated from ovarian tumor (17). Santoro et al. indicated that the chemotherapy response score of neoadjuvant chemotherapy is a prognostic marker involving fibro-inflammatory change in TME (47). Furthermore, Grabosch et al. demonstrated that neoadjuvant chemotherapy with cisplatin enhanced tumor immunogenicity and upregulated PD-L1 expression in ovarian cancer mouse models. We also showed that AZD5153 decreased PD-L1^+^ tumor-induced M2 macrophages *in vitro* (48). Furthermore, AZD5153 resensitizes primary ovary carcinoma and tumor microspheres in ascites to αPD-L1. For these reasons, fresh insight for combining AZD5153 and αPD-L1 is suggested for ovarian cancer. Here, we hypothesize that AZD5153 had synergistic antitumor effects with αPD-L1 by attenuating the expression of PD-L1 on TAMs as well as activating CD8^+^ CTLs. However, further research for the study of the mechanism will be necessary to complete our insights.

In this study, we clarify that AZD5153, as a BRD4 inhibitor, resets TAMs from M2-type to M1-like macrophages through impeding transcriptional binding events for MAF, downregulating the expression of PD-L1 on TAMs, and augmenting activation of CD8^+^ CTLs. It is the first study that illustrates the possibility of AZD5153 functioning as an immune modulator in ovarian cancer which effectively reshapes the tumor-mediated immune suppression microenvironment to a more favorable immune microenvironment that sensitizes the tumor to the effects of immune checkpoint inhibitors Moreover, we provide a future combinational therapeutic strategy likely to increase the population that would benefit from αPD-L1 in ovarian cancer.

## Methods

### Animal Experiments

Female 6-week BALB/c and C57BL/6 mice were purchased from Beijing Huafukang Biotechnology Co., Ltd. Experiments in this study were approved by the Institutional Animal Care and Use Committee at Tongji Medical College of the Huazhong University of Science and Technology.

In the subcutaneous tumor model, each mouse was subcutaneously injected with 5 ^*^ 10^5^ CT26 cells in their right limbs. Visible solid tumors were observed in 1 week. All the mice were divided randomly into two groups, one of which was treated with AZD5153 (0.5 mg/ml) 100 μl/day by gavage while the other was dealt with vehicle for 4 weeks. The volume was recorded every 3 days by vernier calipers and calculated according to the formula *V* = (*L* × *W*^2^)/2 (*L*: length; *W*: width). After the mice were sacrificed, solid tumors were dissociated with a tissue dissociation kit (MACS, USA) for further experiments.

In the intraperitoneal tumor model, each mouse was injected with 5 ^*^ 10^6^ ID8-luc cells. Mice were imaged 3–4 weeks after injection. Mice with positive radiographic signal of intraperitoneal tumor were selected and randomly divided into four groups (*n* = 5). The usage of AZD5153 was the same as above. Anti-PD-L1 (BE0101, clone B7-H1, Bio X Cell) was administered intraperitoneal injection at 200 μg per mouse every 3 days six times in total. Four weeks later, mice were injected with 150 μl of chloral hydrate (5%) and 100 μl of d-luciferin potassium salt (15 mg/ml) into the peritoneum. Images were obtained after 5 min later with the Xenogen VivoVision IVIS Bioluminescent and Fluorescent Imager (PerkinElmer, Waltham, MA). The solid tumors of each mouse were collected and weighed after dissection.

### Cell Lines

Mouse colon carcinoma cell line CT26, leukemia cells in mouse macrophage RAW264.7, human ovarian cancer cell line OVCAR4, and THP-1 (human monocyte cell line) were purchased from ATCC (Manassas, VA, USA). Mouse ovarian epithelial cancer cell line ID8 was obtained from Professor K. Roby (Department of Anatomy and Cell Biology, University of Kansas). CT26 and ID8 were cultured in DMEM supplemented with 10% FBS with penicillin and streptomycin (100 mg/ml). OVCAR4 and THP-1 were maintained in RPMI-1640 with 10% FBS (Gibco, California, USA) with penicillin and streptomycin. Cell cultures were maintained in a humidified atmosphere at 5% CO_2_ at 37°C.

### Cell Experiments

#### Establishment of the TAM Model

##### Polarization-of-m2-type-macrophagesh

RAW264.7 cells were polarized toward the M2 subtype under the stimulation with IL-4 for 24 h in complete RPMI media. For monocyte–macrophage differentiation, THP-1 cells were stimulated with PMA (100 ng/ml) for 48 h (19). Subsequently, THP-1 differentiated macrophages were cultured in complete RPMI media for 6 h and then were treated with IL-4 and IL-13 (20 ng/ml) for 48 h for their polarization toward the M2 subtype. Matured macrophages were harvested and directly used for further experiments.

##### In-vitro-tams-model

We removed the supernatant of ID8 and OVCAR4 cells, washed them with phosphate buffer saline (PBS) three times, and treated them with complete culture medium (CM) for 48 h to obtain mouse or human tumor CM. RAW264.7, a mouse macrophage cell line, was cultured with mouse CM from ID8 cultured for 48 h. Similarly, matured THP-1 was cultured with OVCAR4 supernatant.

#### Isolation of PBMCs and Stimulation of Human Blood Monocytes

Human peripheral blood mononuclear cells (PBMCs) in this article were obtained from five healthy donors and extracted by Ficoll-Paque density gradient separation (density 1.077 g/dl; TBD Science, China, LTS1077). Blood samples were collected in venous blood collection tubes containing EDTA and stored at room temperature. Centrifugation at 1,200 rpm was performed for 10 min to separate cells from plasma. Blood was diluted 1:1 with PBS, added gently to a layer of Ficoll, and centrifuged at 800 g for 25 min at 25°C. The layer of PBMCs is then collected into a new tube and washed twice in PBS through centrifugation (1,200 rpm for 5 min at 25°C. PBMCs were resuspended in serum-free medium RPMI-1640. Monocytes were separated by CD14 Microbeads Ultrapure (Miltenyi Biotec, German, 130-050-201) and then matured with 10 ng/ml of MCSF (PeproTech, USA, 300-25) for 48 h in complete RPMI-1640 with 10% FBS (Gibco, California, USA). Mature macrophages were used for the following experiments.

#### Isolation of BMDMs From Mice

The mice were sterilized with 75% alcohol. The thighbones were completely taken out without any destruction and washed by PBS three times. The bone marrow was washed out by red cell lysate into a clear tube after both sides of the thighbones were cut off and was kept at room temperature for 10 min. The cells was centrifuged and washed by PBS at least two times and resuspended in serum-free medium RPMI-1640. The BMDMs stuck to the surface of the vessels after being planted for 30 min, and the culture medium was then replaced by complete medium for the further experiments.

#### Extraction and Maturation of Human T Lymphocytes

T lymphocytes existed in CD14-negative mononuclear cells separated by CD14 Microbeads Ultrapure (MACS). Cultured with complete RPMI-1640 with 10% FBS (Gibco, California, USA), T lymphocytes were proliferated by human recombinant IL-2 (20 ng/ml) and activated by anti-human CD3/CD28 monoclonal antibody beads for 72 h. Finally, activated T lymphocytes grew in suspension.

#### Transient Transfection of Cells

Induced THP-1 macrophages were transfected according to the procedure of Lipo3000 (Invitrogen, USA) with siRNA (100 μM). Then the cells were cultured for another 48 h for further experiments.

#### AZD5153 Depolarized Tumor-Associated Macrophages

The supernatant of the polarized macrophages mentioned above was removed. The cell plates were washed by PBS three times, and the macrophages were incubated by complete culture medium with 1 μM AZD5153 for 24 h.

### Flow Cytometry

For monocyte and macrophage, sorting cells were resuspended in PBS and stained in a final volume of 100 μl. The following antibodies were used at 1:100 dilutions: F4/80 APC, Gr-1 Percy 5.5, CD206 PE, CD163 PerCy 5.5, CD11b APC (BioLegend). Cells were incubated in the dark for 30 min at 4°C. For T lymphocytes, sorting cells were dealt with the same methods mentioned above, using these antibodies at 1:100 dilutions: CD3 APC, CD8 PE, or IFN-γ PerCy 5.5 (BioLegend). Cells were incubated in the dark for 30 min at 4°C. After being washed with PBS, cells were filtered and resuspended in PBS before analysis.

When it comes to cell proliferation, enriched T lymphocytes were labeled by CFSE (Sigma-Aldrich) for 10 min, and labeling by FBS was stopped. Fifteen minutes later, cell suspension was centrifuged and washed with PBS five times. Finally, labeled T lymphocytes were resuspended into an RPMI-1640 medium containing 10% FBS. T-cell proliferation was measured for CFSE dilution 0, 6, 12, 24, and 48 h later by flow cytometric analysis. Data were acquired by Beckman Coulter and analyzed with FlowJo software.

### Real-Time qPCR

Total RNA (2 μg) was extracted with TRIzol reagent (Invitrogen, USA) and reverse-transcribed into cDNA. The cDNA was amplified on an RT-PCR System (Bio-Rad). The mRNA level was normalized by β-actin. Primers for qPCR were shown in Supplementary Table 1.

### Chromatin Immunoprecipitation–qPCR

Chromatin immunoprecipitation (ChIP) assays were performed with a ChIP kit (CST, USA) as described in the instructions. Cells were crosslinked with 1% formaldehyde. The nuclei were isolated and subjected to sonication for chromatin fragmentation after cell lysis. Sheared chromatin was diluted in the buffer and distributed into aliquots for immunoprecipitation. Chromatin samples were incubated with overnight rotational incubation at 4°C after adding anti-BRD4 antibody (NOVAS). Antibody–chromatin complexes were captured by magnetic protein A/G beads. Purified DNAs were quantified by real-time PCR. All primers were listed in the Supplementary Table 1.

### ELISA

THP-1 and PBMCs were cultured according to the cell culture methods mentioned above. The culture medium derived from OVCAR4 cells was applied for induction of THP-1 and PBMCs. Human recombination cytokines IL-10 and IL-12 were detected for CM-induced macrophages treated with and without AZD5153. Enzyme-linked immunosorbent assay (ELISA) was performed according to standard procedures. Human IL-12 and IL-10 ELISA kits were purchased from Neobioscience (Shenzhen, China).

### 3-D Culture for Tumor Microspheres

AIM Biotech 3-D Cell Culture Chip was utilized for 3-D culture (49). Human solid tumors were digested by a tissue dissociation kit (MACS, USA). The tumor tissue was cut into pieces and transferred into C tubes (Miltenyi Biotec) containing enzymes H, R, and A. Mechanical dissociation was accomplished according to three steps on the gentleMACS Dissociator. The C tube was rotated for 30 min at 37°C after the first and second steps. Following tumor dissociation, the samples were filtrated at a diameter between 40 and 100 μm. Microsphere suspension consisting of 10 μl of cell suspension above, 143 μl of rat tail collagen IV, 19 μl of PBS, 23 μl of H_2_O, and 5 μl of 0.5 M NaOH was mixed, and every 10 μl of suspension was pipetted into one gel channel. After culturing 3-D culture chips in the 37°C thermostatic incubator for 30 min, 120 μl of complete culture medium was pipetted into each material channel. Microspheres were cultured for 24 h at 37°C thermostatic incubator for further experiments.

### GEO Dataset Analysis and GSEA Analysis

The gene expression dataset of mouse PBMC was taken from the NCBI GEO database (http://www.ncbi.nlm.nih.gov/geo/). Samples included were wild-type or BRD4-knockout mice stimulated with IL-4. The following information was also extracted from the study: GEO accession number (GSE104643), sample type, and gene expression data.

For GSEA analysis, the expression value of each gene was replicated twice and was normalized using the limma package of the R platform (version 3.5.1.; www.r-project.org). The biological pathway named COATES_MACROPHAGE_M1_VS_M2_DN was explored with GSEA using data derived from the Molecular Signatures Database of c2. *P* < 0.05 were determined to confer statistical significance.

### Statistical Analysis

Statistical analysis was performed using GraphPad Prism 7.0. Results were presented as mean ± SEM, and statistical significance was examined by an unpaired Student's *t*-test for two groups and by one-way ANOVA for multiplicity, calculated by SPSS (Version 23.0) software. A *P* < 0.05 was considered statistically significant.

## Data Availability Statement

The raw data supporting the conclusions of this article will be made available by the authors, without undue reservation, to any qualified researcher.

## Ethics Statement

Animal experiments in this study were approved by the Institutional Animal Care and Use Committee at Tongji Medical Collage of Huazhong University of Science and Technology. The studies involving human participants were reviewed and approved by Tongji Medical College, Huazhong University of Science and Technology. The patients/participants provided their written informed consent to participate in this study.

## Author Contributions

XL and YF did the Western Blot, ELISA, and RT-PCR assays. BY, EG, YW, and JHua did the animal assays. XZ and RX did the cell culture. BW, JHu, and KL collected clinical specimens. XL and YF were responsible for data analysis. XL and YF were responsible for data disposal. CS, GC, XL, and YF wrote the article. GC and CS designed the experiments.

### Conflict of Interest

The authors declare that the research was conducted in the absence of any commercial or financial relationships that could be construed as a potential conflict of interest.

## Supplementary Material

Supplementary Figure 1 Conditional medium of ovarian cancer cells (CM) induced M2 type macrophages *in vitro*. **(A)** qPCR evaluation of ARG1 andMRC1 levels in THP-1 and RAW264.7 macrophages with induction of cytokines or CM. THP-1 is induced by human recombinant IL-4 and IL-13, and RAW264.7 is induced by mouse recombinant IL-4 for 24 h. **(B)** Expression of cytokines related to inflammation detected by RT-PCR in cytokines-induced macrophages and CM-induced macrophages. Left: experiment for human macrophages cell line THP-1. Right: experiment for mouse macrophages cell line RAW264.7 (*n* = 3). Data represent Mean ± SEM. ^*^Referring to the difference between the column and the relative vehicle, *p* < 0.05.

Supplementary Figure 2 Pro-inflammatory cytokines expressed in AZD5153-treated TAMs. **(A)** expression of IFN-γ and TNF-α analyzed by qPCR for CM-induced human macrophages cell line THP-1 treated with and without AZD5153. **(B)** expression of IFN-γ and TNF-α analyzed by qPCR for CM-induced mouse macrophage cell lines RAW264.7.

Supplementary Figure 3 RT-PCR for AZD5153 treated M2-type macrophage with three different normalizers. **(A)** The mouse macrophage cell line RAW264.7. **(B)** The human macrophage cell line THP-1. **(C)** The BMDMs extracted from mice (*n* = 3). Data represent Mean ± SEM. ^*^*p* < 0.05.

Supplementary Figure 4 AZD5153 showed synergy with anti-PD-L1 *in vivo*. **(A)** The diagrammatic drawing of solid tumors in each group. **(B)** Weight of the ID8 mouse models during the 4-week-long therapy. **(C)** Tumor microspheres isolated from carcinoma blocks of an untreated ovarian cancer patient in 3-D microfluidic chips. Left: diagram of microspheres under treatment with no drug, single drug (AZD5153 or αPD-L1), and combinational strategy. Right: bar chart demonstrating the percentage of live and dead microspheres (*n* = 3). Data represent mean ± SEM. ^*^*P* < 0.05.

Supplementary Table 1 The primer sequences for RT-PCR and CHIP-PCR.

## Abbreviations

HGSOC: high-grade serous ovarian cancer
TME: tumor microenvironment
TAM: tumor-associated macrophages
CTLs: cytotoxic T lymphocytes
BET: bromodomain and extraterminal
CM: conditional medium
TFBS: transcription factor binding site.
