# Supplementary figures and images for: BRD4 Inhibition by AZD5153 Promotes Antitumor Immunity via Depolarizing M2 Macrophages
Source: Front Immunol. 2020 Feb 28;11:89. doi: 10.3389/fimmu.2020.00089 (PMC7058627; doi:10.3389/fimmu.2020.00089)

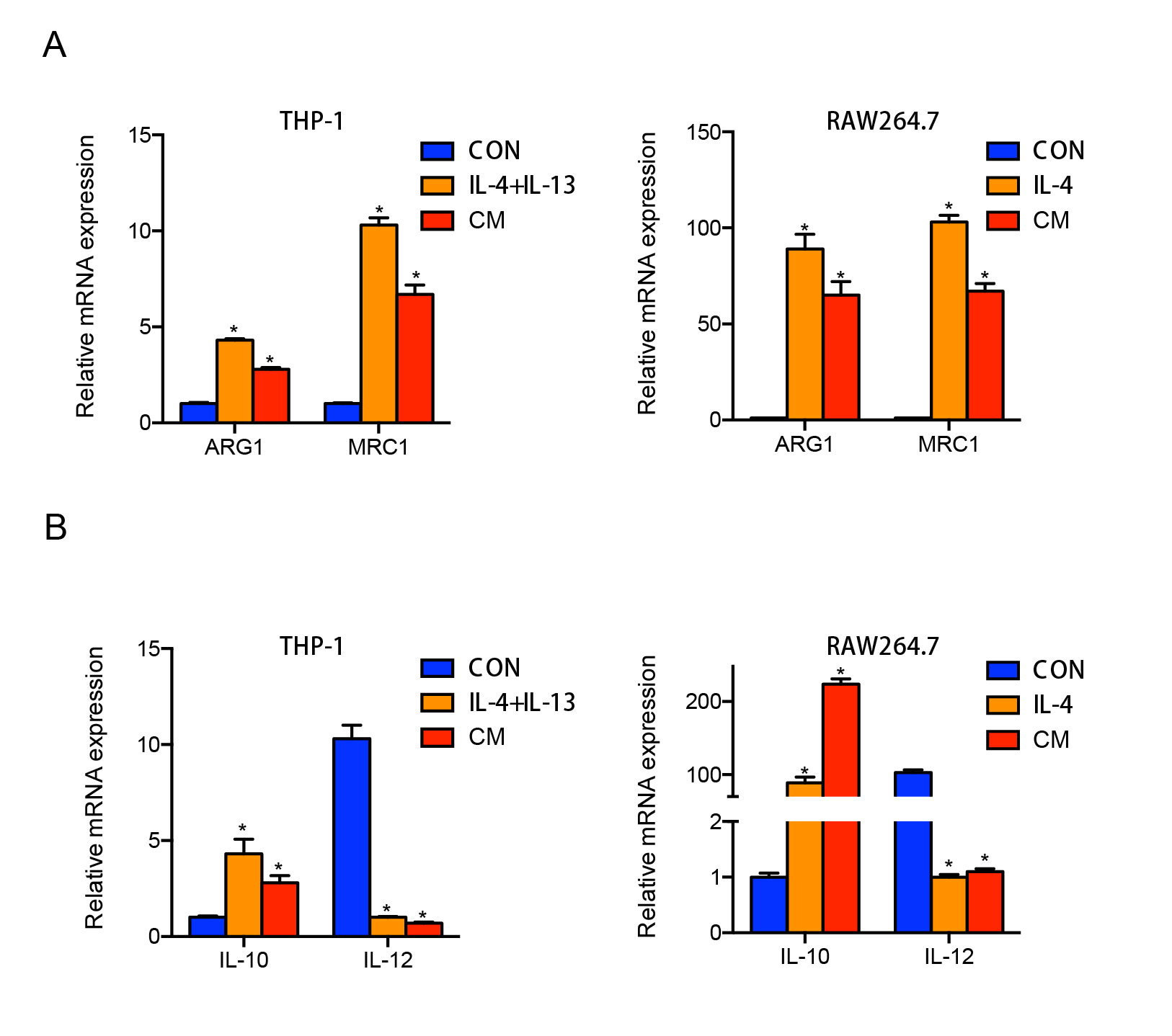

Supplement: Supplementary Figure 1 — Conditional medium of ovarian cancer cells (CM) induced M2 type macrophages in vitro. (A) qPCR evaluation of ARG1 andMRC1 levels in THP-1 and RAW264.7 macrophages with induction of cytokines or CM. THP-1 is induced by human recombinant IL-4 and IL-13, and RAW264.7 is induced by mouse recombinant IL-4 for 24 h. (B) Expression of cytokines related to inflammation detected by RT-PCR in cytokines-induced macrophages and CM-induced macrophages. Left: experiment for human macrophages cell line THP-1. Right: experiment for mouse macrophages cell line RAW264.7 (n = 3). Data represent Mean ± SEM. *Referring to the difference between the column and the relative vehicle, p < 0.05. [file Image_1.jpg]

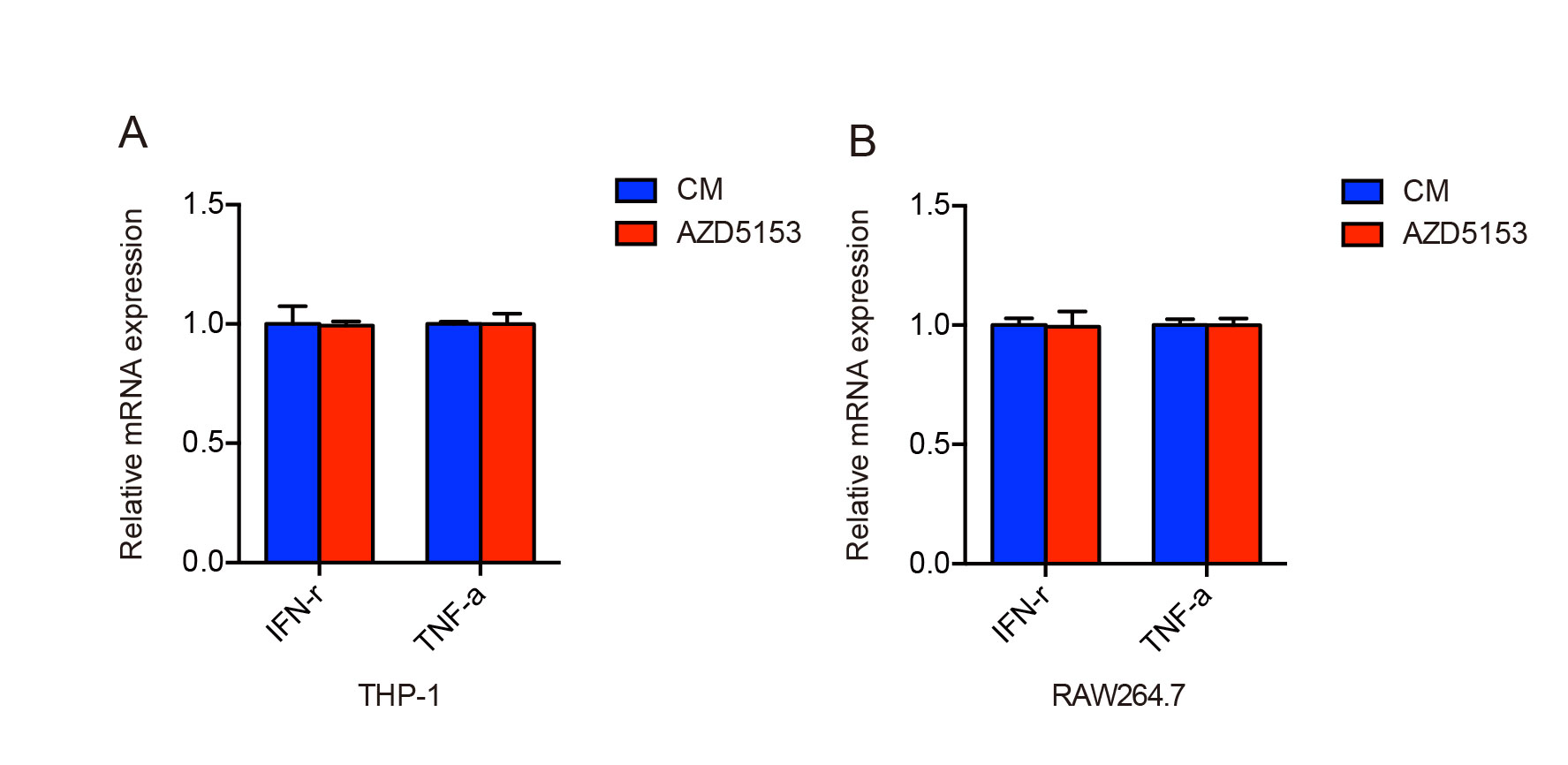

Supplement: Supplementary Figure 2 — Pro-inflammatory cytokines expressed in AZD5153-treated TAMs. (A) expression of IFN-γ and TNF-α analyzed by qPCR for CM-induced human macrophages cell line THP-1 treated with and without AZD5153. (B) expression of IFN-γ and TNF-α analyzed by qPCR for CM-induced mouse macrophage cell lines RAW264.7. [file Image_2.jpg]

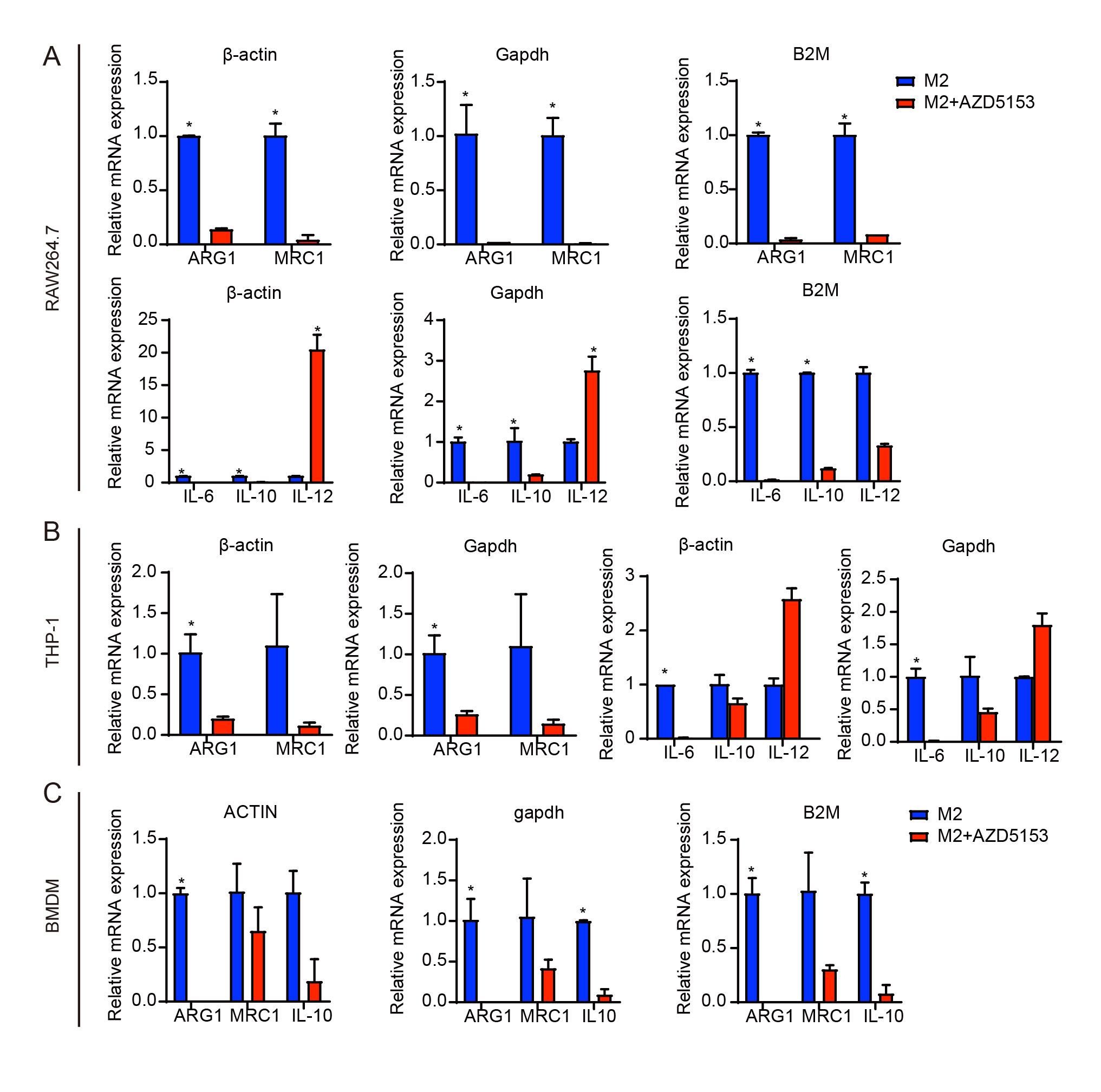

Supplement: Supplementary Figure 3 — RT-PCR for AZD5153 treated M2-type macrophage with three different normalizers. (A) The mouse macrophage cell line RAW264.7. (B) The human macrophage cell line THP-1. (C) The BMDMs extracted from mice (n = 3). Data represent Mean ± SEM. *p < 0.05. [file Image_3.jpg]

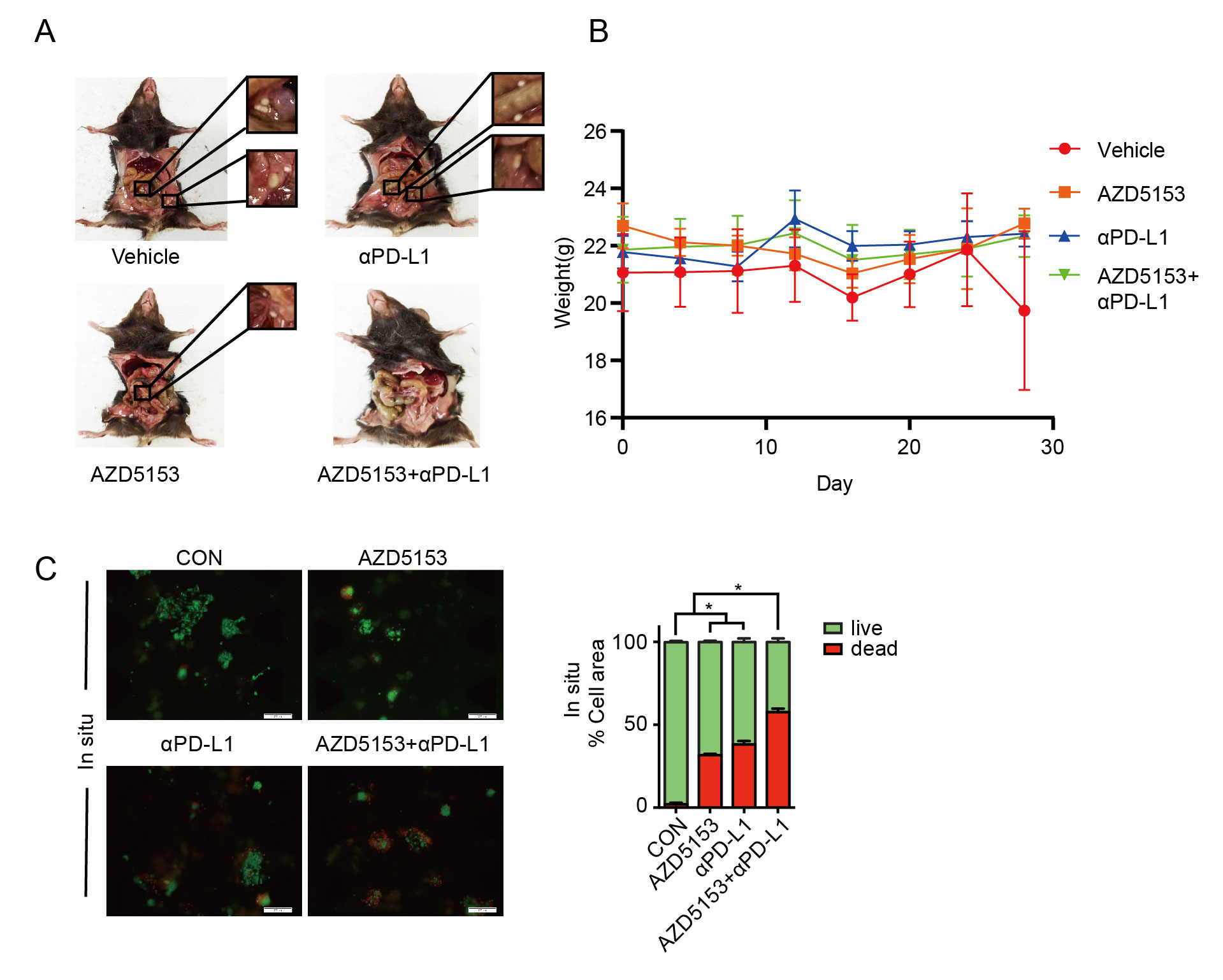

Supplement: Supplementary Figure 4 — AZD5153 showed synergy with anti-PD-L1 in vivo. (A) The diagrammatic drawing of solid tumors in each group. (B) Weight of the ID8 mouse models during the 4-week-long therapy. (C) Tumor microspheres isolated from carcinoma blocks of an untreated ovarian cancer patient in 3-D microfluidic chips. Left: diagram of microspheres under treatment with no drug, single drug (AZD5153 or αPD-L1), and combinational strategy. Right: bar chart demonstrating the percentage of live and dead microspheres (n = 3). Data represent mean ± SEM. *P < 0.05. [file Image_4.jpg]
